# Marker-based versus model-based radiostereometric analysis of total knee arthroplasty migration: a reanalysis with comparable mean outcomes despite distinct types of measurement error

**DOI:** 10.1080/17453674.2019.1605692

**Published:** 2019-04-24

**Authors:** Koen T van Hamersveld, Perla J Marang–van de Mheen, Lennard A Koster, Rob G H H Nelissen, Sören Toksvig-Larsen, Bart L Kaptein

**Affiliations:** a Department of Orthopaedics, Leiden University Medical Center, Leiden, the Netherlands;; b Department of Biomedical Data Sciences, Leiden University Medical Center, Leiden; the Netherlands;; c Department of Orthopaedics, Hässleholm Hospital, Hässleholm, Sweden and Department of Clinical Sciences, Lund University, Lund, Sweden

## Abstract

Background and purpose — Pooling data of studies evaluating total knee arthroplasty migration using radiostereometric analysis (RSA) may be compromised when the RSA method used would influence estimated differences between groups. We therefore reanalyzed a marker-based RSA study with model-based RSA to assess possible limitations of each RSA method, including insert micromotions in modular TKA and their effect on estimated group differences.

Patients and methods — All patients had received a cemented Triathlon implant (Stryker, Mahwah, NJ, USA) with either an all-polyethylene (n = 29) or a metal-backed (n = 28) tibial component. The latter group was reanalyzed with model-based RSA. Precision of each RSA method was calculated using double examinations. Bland–Altman plots were constructed to determine the limits of agreement between the 2 RSA methods. Polyethylene insert micromotion was quantified by measuring migration with respect to the metal tray. Finally, analyses of the original study were repeated with the model-based RSA results.

Results — Systematic differences were found in translations between marker-based and model-based RSA as a result of different reference origins being used for migration calculations. Micromotions of the polyethylene insert within the metal tray were negligibly small. Mean migration results were comparable between marker-based and model-based RSA when using the same reference origin, even though conclusions on individual patients may differ between RSA methods due to various types of measurement error (e.g., marker occlusion and model-fit inaccuracies).

Interpretation — At least for the studied TKA design, pooling mean migration data of different RSA methods appears justified. For translations, however, adjustments should be made to correct for differences in reference origin. Migration patterns of individual patients may differ as a result of distinct types of measurement error.

Due to the high accuracy and precision of radiostereometric analysis (RSA), late loosening of new implants can already be predicted with 2-year RSA results on small patient numbers (Ryd et al. 1995, Valstar et al. 2005, Nelissen et al. 2011). RSA requires the bone and prosthesis to be accurately defined in 3 dimensions, usually achieved by inserting tantalum markers in the bone and by attaching or inserting markers (in)to the prosthesis (i.e., marker-based RSA). Prosthesis markers are generally inserted during surgery in the polyethylene of the implant (Kaptein et al. 2007). Alternatively, in model-based RSA the need for prosthesis markers is eliminated by matching a virtual projection of a 3D model with the contours of the radiographic projection of the implant (Kaptein et al. 2003). Results of model-based RSA are suggested to be comparable with conventional marker-based methods on a group level (Pijls et al. 2018), but direct comparisons on individual patient data are scarce (Kaptein et al. 2007, Hurschler et al. 2009).

We recently published the 2-year results of a randomized controlled trial (RCT) on implant migration of cemented metal-backed versus all-polyethylene tibial components in total knee arthroplasty (TKA) using the Triathlon TKA system (Stryker, Mahwah, NJ, USA) (van Hamersveld et al. 2018). Higher migration was found after 2 years for the metal-backed components, even though the difference was small. However, as migration measurements were based on markers inserted in the polyethylene, apparent migration of the modular metal-backed components may partly result from micromotion of the polyethylene insert with respect to the metal tray, a phenomenon that has been shown to occur in older fixed-bearing designs (Nilsson et al. 2003, Hansson et al. 2005).

In this study, we reanalyzed the metal-backed components with model-based RSA to eliminate any influence of modularity on migration results and thus investigate whether methodological differences between RSA methods would affect migration results. Second, we quantified movements of the polyethylene insert within the locking mechanism of the metal tray. Finally, we investigated whether the use of model-based RSA would result in different conclusions of the RCT as compared with the marker-based results. 

## Patients and methods

Full details of the original RCT regarding patients, randomization, follow-up, prosthesis, and surgical techniques have been described previously (van Hamersveld et al. 2018). Briefly, 2 surgeons implanted cemented, condylar-stabilizing, cruciate-retaining Triathlon total knee prostheses with either all-polyethylene (n = 29) or modular fixed-bearing metal-backed tibial components (n = 30). The metal tray was designed with a full peripheral capture locking mechanism and an anti-rotational central island (Łapaj et al. 2017). 2 patients with metal-backed components were analyzed with model-based RSA in the original RCT due to polyethylene marker occlusion, which precluded marker-based measurements. Hence, no marker-based results were available for comparison and these were thus excluded in the present study.

### Radiostereometric analysis

**Figure 1.**
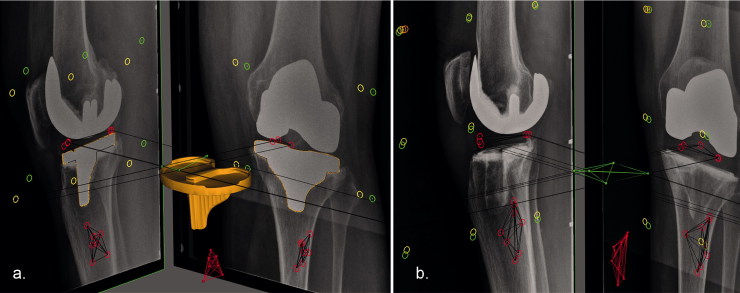
RSA images showing the biplanar (lateral and anteroposterior) views with the polyethylene markers and tibial bone markers encircled in red, the fiducial markers in yellow, and the control markers in green. (a) Only 3 of 5 polyethylene markers are visible due to over-projection of 2 markers, in most cases, by the femoral component, which may reduce or invalidate the marker-based accuracy of the RSA measurement. However, migration can also be measured by fitting a model using the contours of the metal-backed tibial component as shown in orange. (b) Migration of the radiolucent all-polyethylene tibial component can only be measured with marker-based RSA.

**Figure 2.**
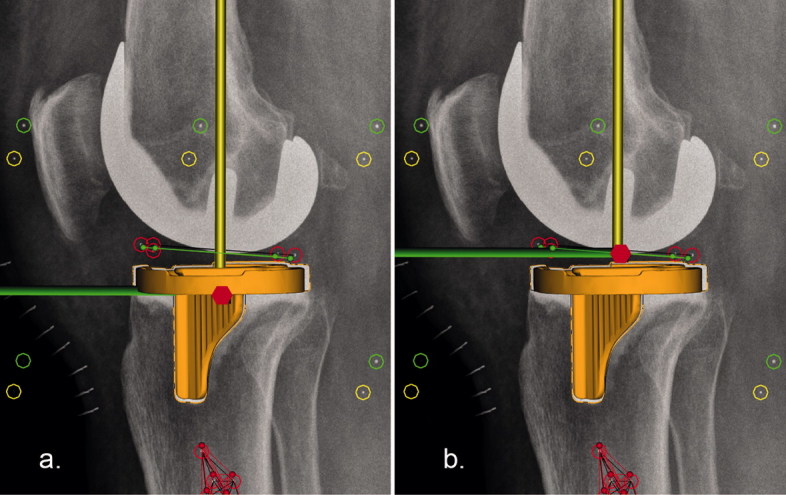
Lateral views showing the different reference origins (center of migrating model at reference time point T0) in (a) model-based and (b) marker-based RSA. The longitudinal axis is represented by the yellow line, the sagittal axis by the green line, and the red hexagon represents the origin. To fully compare model-based and marker-based RSA data using the same reference origin, a second model-based analysis was performed with the reference origin fixed in the center of the polyethylene markers as shown in b.

The first RSA examination, performed on the first postoperative day, served as the reference for the migration measurements. Subsequent examinations were performed at 3 months, 1 year, and 2 years after surgery. RSA radiographs were performed in supine position with the knee in a biplanar calibration cage (cage 10, RSA Biomedical, Umeå, Sweden) and analyzed using Model-based RSA software version 4.1 (RSAcore, LUMC, Leiden, the Netherlands). For marker-based RSA analysis 5 tantalum markers (0.8 mm in diameter) were inserted during surgery, after drilling appropriate holes, at standardized positions in the polyethylene of both tibia designs. 2 markers were placed posteriorly, 2 anteromedially/anterolaterally, and 1 anteriorly. The number of markers available for migration calculations could differ over time due to marker occlusion (Figure 1a). Marker-based results of the metal-backed group were based on all 5 polyethylene markers in only 3 patients. As a result of marker occlusion in 1 or more follow-up moments, marker-based results were based on 4 polyethylene markers in 8 patients and on 3 markers in 17 patients. Model-based reanalysis was performed only in the metal-backed group, as the all-polyethylene components are radiolucent (Figure 1b). In the RSA analysis of the original report, a triangulated surface model (from reversed engineering, reduced to 5,000 triangles) was added for the tibial component and its virtual projections were matched with the contours of the radiographic projection of the implant. All other aspects of the analysis, such as insert markers, bone markers, and calibration markers, remained unchanged. Migration of the 28 metal-backed tibial components, by means of the 3D surface model, was calculated twice: with the reference origin for migration calculations (1) in the geometric center of the model, which is the standard position for model-based RSA analysis, and (2) in the geometric center of the polyethylene markers, which is the standard position for marker-based RSA analysis (Figure 2). In addition, migration of the polyethylene insert markers was determined to assess whether the insert moved with respect to the metal tray. Lastly, method 2 allowed us to compare model-based metal-backed results with marker-based all-polyethylene results using the same reference origin. The precision of each RSA method was determined by means of double examinations at 1-year follow-up. The precision is expressed as the upper limits of the 95% confidence interval (CI) around zero motion (ISO 16087:2013(E) 2013). The primary outcome measure used in the original report is the maximum total point motion (MTPM), which is the length of the translation vector of the marker that moved the most. For model-based RSA, MTPM is the length of the translation vector of the point on the model that moved the most. We also report the number of individual components showing “continuous migration,” defined by Ryd et al. (1995) as an increase in MTPM of ≥ 0.2 mm in the second postoperative year. The limits of marker stability (mean error) and scatter values (condition number) were set at 0.35 mm and 120, respectively, complying with the RSA guidelines (Valstar et al. 2005).

### Statistics

We first estimated differences in model-based analyses with 2 different reference origins, i.e., the reference origin in the geometric center of the model versus the geometric center of the polyethylene markers, using regression analysis. Bland–Altman plots were constructed to determine the limits of agreement between the two RSA methods (Bland and Altman 1986). The limits of agreement, defined as the mean ± 1.96 × SD, should be within ± 0.5 mm of translation or ± 0.8° of rotation for the measures to be considered equivalent. These thresholds were chosen as these are considered the smallest values of clinically relevant early migration when used as a predictor of aseptic loosening (Hurschler et al. 2009, Gudnason et al. 2017, Pijls et al. 2018). Boxplots were constructed to investigate micromotion of the polyethylene markers with respect to the metal tray along and about each orthogonal axis. Finally, an identical linear mixed-effects model as described in the original report (van Hamersveld et al. 2018) was used to analyze differences in migration between (model-based) metal-backed and (marker-based) all-polyethylene components while using the same reference origin (center of the polyethylene markers). As in the original report, log-transformation of outcome measures was applied when necessary to obtain normal distributions, and the same sensitivity analysis was performed given the unevenly distributed baseline characteristics sex and surgeon as possible confounders by adding these variables to the linear mixed-effects model (van Hamersveld et al. 2018). Significance was set at p < 0.05 (IBM SPSS Statistics 24.0; IBM Corp, Armonk, NY, USA).

### Ethics, registration, funding, and potential conflicts of interest

The original study was approved by the Regional Ethical Review Board in Lund (entry no. 2013/434) and registered at isrctn.com (ID: ISRCTN04081530). All patients gave informed consent. The costs of the RSA radiographs made for the original study were supported by Stryker. The sponsor did not take part in the design, conduct, analysis, or interpretations stated in both the previous and current manuscript. The authors declare no competing interests. 

## Results

Double examinations were performed in 21 metal-backed components at 1-year follow-up to determine the precision of the RSA measurements. Model-based results were less precise in rotations, especially about the longitudinal axis (Table 1).

**Table 1.** Precision of RSA measurements (upper limits of the 95% CI around zero motion unless otherwise stated)

Group RSA method	Translations (mm)			Rotations (°)			MTPM (mm)	
	Transverse	Longitudinal	Sagittal	Transverse	Longitudinal	Sagittal	Mean	Upper limit of CI
All-polyethylene (n = 26 double examinations)								
Marker-based	0.11	0.15	0.09	0.17	0.11	0.11	0.14	0.14
Metal-backed (n = 21 double examinations)								
Marker-based	0.07	0.11	0.13	0.08	0.14	0.11	0.12	0.11
Model-based	0.08	0.11	0.13	0.19	0.64	0.15	0.25	0.32
Polyethylene micromotion	0.06	0.06	0.11	0.14	0.68	0.16	0.19	0.30

### Marker-based versus model-based RSA

**Figure 3.**
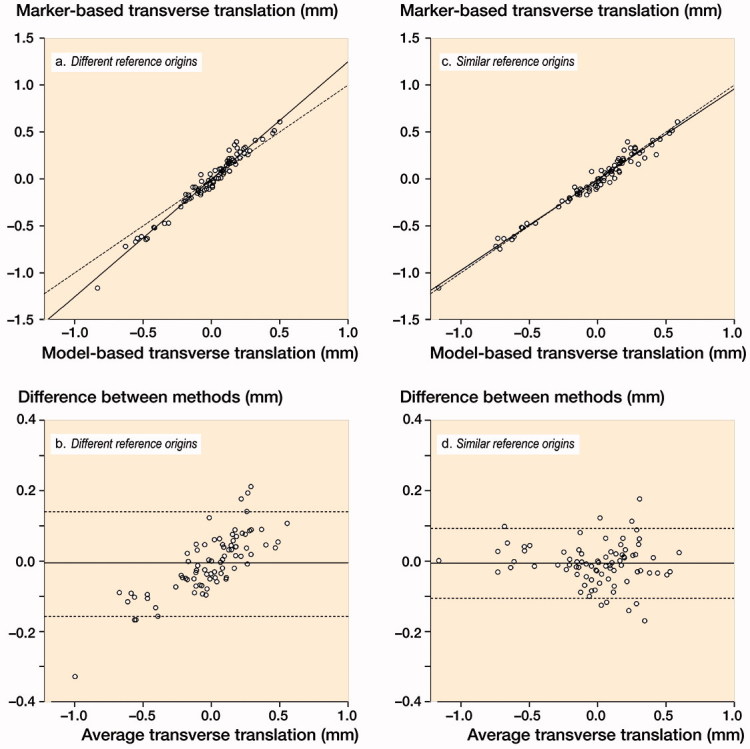
Scatter-plots showing (a) that marker-based transverse translation values are generally larger than model-based values due to the difference in position of the geometric center (which is either in the geometric center of the markers inserted in the polyethylene or in the geometric center of the model), also indicated (in b) by the proportional bias observed in the Bland–Altman plot (i.e., the difference between methods is proportional to the level of the measured variable) (Ludbrook 1997). (c) If model-based analysis is performed with the reference origin fixed at the geometric center of the polyethylene markers, results are nearly identical between methods, as also indicated (in d) by the absence of proportional bias and the small limits of agreement in the Bland–Altman plot. Solid lines in a and c: regression line. Dashed lines in a and c: line of equality. Solid horizontal lines in b and d: mean of differences. Dashed horizontal lines in b and d: 95% limits of agreement.

Regression analysis revealed that with (1) routine model-based RSA versus (2) model-based RSA with the reference origin in the geometric center of the polyethylene markers, the transverse, longitudinal, and sagittal translations were overestimated by 29% (CI 25–32), 7% (CI 0–13) and 26% (CI 24–28), respectively (illustrated for transverse translations in Figures 3a and 3b). As expected (for mathematical reasons, see Appendix), rotations and MTPM values were not influenced by the position of the reference origin and therefore identical between both model-based analyses. For fair comparison of marker-based and model-based translations, the reference origin for the model-based analysis was thus fixed at the geometric center of the polyethylene markers for the remaining analyses described below. This resolved the proportional bias (shown in Figure 3b and absent in Figure 3d) (Ludbrook 1997).

Comparing marker-based with model-based RSA, translations showed small limits of agreement indicating that both methods can be used interchangeably (Table 2). The limits of agreement for the rotations and MTPM were larger, especially for rotations about the longitudinal axis (Table 2).

**Table 2.** Differences between marker-based and model-based translations and rotations with the reference origin fixed at the geometric center of the polyethylene markers

	Translations (mm)			Rotations (°)			MTPM
Factor	Transverse	Longitudinal	Sagittal	Transverse	Longitudinal	Sagittal	(mm)
Mean (SD)	–0.01 (0.05)	–0.03 (0.05)	–0.05 (0.10)	–0.02 (0.13)	0.09 (0.29)	–0.06 (0.18)	–0.03 (0.21)
95% CI **^a^**	–0.11 to 0.09	–0.12 to 0.07	–0.25 to 0.16	–0.28 to 0.24	–0.48 to 0.66	–0.41 to 0.29	–0.45 to 0.39

**^a^**The values represent the limits of agreement (interchangeability) between the 2 methods (Bland and Altman) and are based on all (n = 28) patients.

### Micromotion of the polyethylene insert with respect to the metal tray

**Figure 4.**
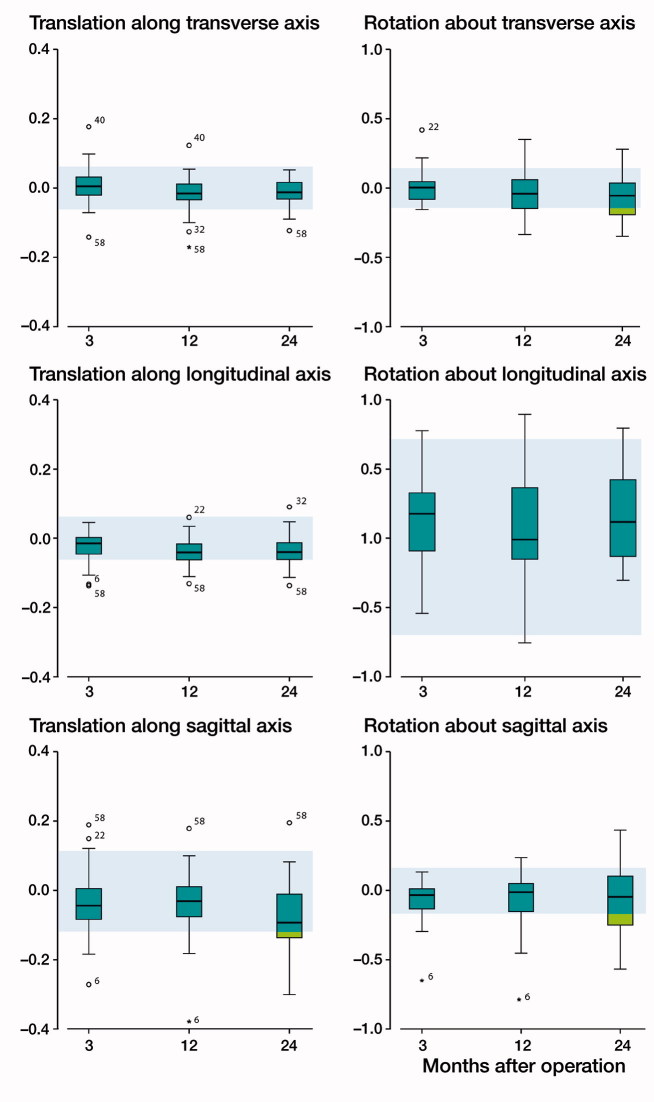
Box-and-whisker plots showing the polyethylene insert translations and rotations with respect to the metal tray at each follow-up (n = 28). The line in boxes indicate group median, the box the interquartile range (IQR); the whiskers the maximum values and outliers are depicted as circles (> 1.5 × IQR) and stars (> 3 × IQR). Shaded blue areas represent the 95% confidence intervals of zero motion (i.e., RSA precision, determined with double examinations), numbers of the outliers are patient study numbers.

Boxplots were constructed to investigate micromotion of the polyethylene insert with respect to the metal tray along and about each orthogonal axis at 3, 12, and 24 months’ follow-up (Figure 4). The majority of the measurements were within the 95% confidence interval of zero motion (i.e., the precision of the RSA method, indicated by the shaded areas in Figure 4) and group median values did not appear to increase over time. A few outliers depicted in Figure 4 were evaluated to determine the nature of the extreme values, all of which were found to be due to measurement error as a result of instability or occlusion of the polyethylene markers. The error of patient 6 was due to one polyethylene marker moving posteriorly close to the periphery of the drilled hole where it was inserted (resulting in a mean error between 0.31 and 0.33 at 3, 12, and 24 months, close to the limit of 0.35). This marker stabilized within 3 months, as the polyethylene micromotion values were close to zero when 3 months’ follow-up was taken as the reference (mean error between 0.02 and 0.03 at 12 and 24 months). A similar cause was found in the analysis of patient 58, but in this case 2 anterior markers moved anteriorly; results were also close to zero when 3 months’ follow-up was taken as the reference. In the analysis of patient 22, patient 32, and patient 40, only 3 polyethylene markers were available of which 1 was partly occluded in 1 or more follow-up moments by either the tibial component or by another marker; slightly adjusting the position of these markers resulted in values close to zero in all directions.

### Change in results of original trial

**Figure 5.**
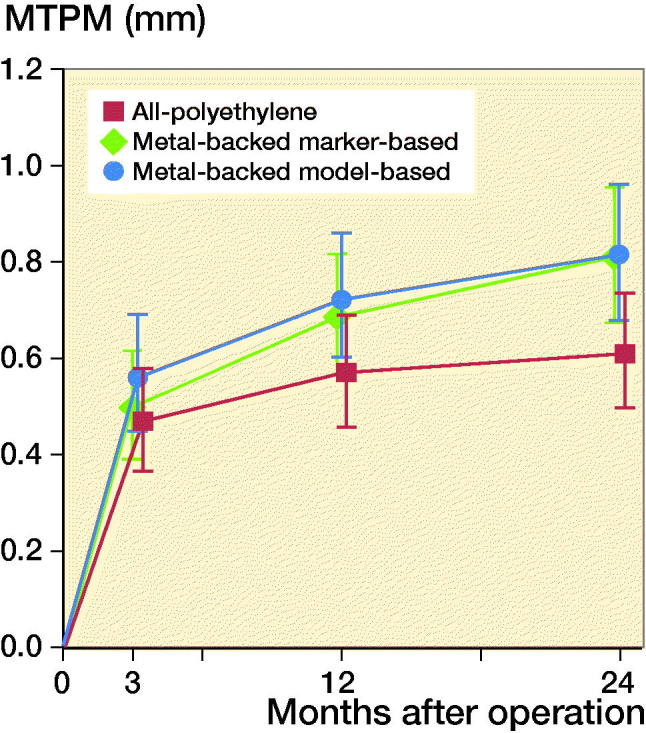
RSA analysis results of maximum total point motion (MTPM). The mean and 95% confidence interval for the metal-backed group is shown for both the marker-based (greeen line) as well as the model-based (blue line) analysis.

When repeating the analysis of the primary outcome (MTPM after 2 years of follow-up) of the original report (van Hamersveld et al. 2018) with the model-based migration values, comparable group differences were found: the all-polyethylene group had an MTPM (CI) of 0.61 (0.49–0.74) versus 0.81 (0.68–0.95) for the marker-based metal-backed group; and versus 0.82 (0.68–0.96) for the model-based metal-backed group (Figure 5).

In the original paper, continuous migration of ≥ 0.2 mm in MTPM in the second postoperative year was seen in 4 components in both groups. These 4 individual components of the metal-backed group showed similar migration patterns using model-based analysis (i.e., continuous migration in the second postoperative year). However, 2 additional metal-backed components showed continuous migration based on the model-based analysis. In both cases, the increase in MTPM in the second postoperative year was likely the result of a sudden increase in rotation about the longitudinal axis due to model-fit inaccuracies, as all other parameters remained stable (data not shown).

The other RSA parameters showed comparable between-group results when repeating the analysis with model-based migration values, except for translations along and rotations about the longitudinal (y-)axis, again, due to model-fit inaccuracies (Table 3, see Supplementary data).

**Table 3.** RSA migration analysis of mean absolute translation and rotation along and about each orthogonal axis (log-values are back-transformed in the original scale)

	All-polyethylene mean (95% CI) (n = 29)	Metal-backed marker-based mean (95% CI) (n = 28)	Metal-backed model-based mean (95% CI) (n = 28)
Translation along transverse axis (mm)			
3 months	0.14 (0.09–0.20)	0.21 (0.15–0.27)	0.20 (0.15–0.27)
1 year	0.14 (0.09–0.20)	0.21 (0.16–0.27)	0.21 (0.16–0.27)
2 years	0.19 (0.14–0.25)	0.26 (0.20–0.32)	0.26 (0.20–0.32)
Translation along longitudinal axis (mm)			
3 months	0.12 (0.08–0.15)	0.11 (0.08–0.15)	0.12 (0.08–0.15)
1 year	0.13 (0.09–0.16)	0.13 (0.09–0.16)	0.15 (0.12–0.19)
2 years	0.10 (0.07–0.14)	0.15 (0.11–0.18)	0.17 (0.14–0.21)
Translation along sagittal axis (mm)			
3 months	0.19 (0.11–0.27)	0.22 (0.14–0.31)	0.24 (0.16–0.33)
1 year	0.24 (0.16–0.33)	0.38 (0.29–0.47)	0.38 (0.29–0.48)
2 years	0.25 (0.17–0.34)	0.44 (0.35–0.55)	0.43 (0.34–0.53)
Rotation about transverse axis (°)			
3 months	0.38 (0.27–0.49)	0.23 (0.14–0.34)	0.25 (0.15–0.35)
1 year	0.48 (0.37–0.61)	0.38 (0.27–0.49)	0.40 (0.30–0.52)
2 years	0.47 (0.36–0.59)	0.47 (0.35–0.59)	0.45 (0.33–0.57)
Rotation about longitudinal axis (degrees)			
3 months	0.18 (0.11–0.25)	0.19 (0.13–0.27)	0.29 (0.22–0.38)
1 year	0.20 (0.13–0.28)	0.24 (0.17–0.31)	0.38 (0.30–0.47)
2 years	0.20 (0.13–0.27)	0.28 (0.20–0.35)	0.41 (0.32–0.50)
Rotation about sagittal axis (°)			
3 months	0.26 (0.18–0.33)	0.24 (0.16–0.32)	0.21 (0.14–0.28)
1 year	0.32 (0.25–0.40)	0.28 (0.20–0.36)	0.24 (0.16–0.31)
2 years	0.34 (0.26–0.42)	0.33 (0.25–0.41)	0.25 (0.18–0.33)

In this trial, 2 surgeons performed the surgeries. When stratifying the results by surgeon as performed in the post hoc sensitivity analysis of the original report, the observed difference in MTPM in favor of the all-polyethylene design was smaller and not statistically significant. Repeating this sensitivity analysis with the model-based measurements resulted in similar conclusions (Table 4, see Supplementary data).

**Table 4.** Adjusted RSA migration analysis of log-transformed maxi-mum total point motion (logMTPM)

	Mean difference in logMTPM between groups (95% CI)			
	Marker-based **^a^**		Model-based **^b^**	
Treatment effect (reference: all-polyethylene)				
3 months	–0.007 (–0.049 to 0.036)		0.013	(–0.031 to 0.057)
1 year	0.014	(–0.029 to 0.057)	0.025	(–0.019 to 0.069)
2 years	0.030	(–0.013 to 0.074)	0.038	(–0.007 to 0.083)
Sex effect (reference: male)				
3 months	0.008	(–0.043 to 0.045)	0.002	(–0.044 to 0.047)
1 year	0.017	(–0.027 to 0.062)	0.011	(–0.034 to 0.057)
2 years	0.026	(–0.020 to 0.068)	0.031	(–0.015 to 0.077)
Surgeon effect (reference: surgeon 1)				
3 months	0.083	(0.040 to 0.126)	0.077	(0.033 to 0.121)
1 year	0.113	(0.071 to 0.156)	0.099	(0.055 to 0.143)
2 years	0.132	(0.089 to 0.174)	0.114	(0.070 to 0.158)

^a^All-polyethylene (n = 29) versus marker-based metal-backed (n = 28).

^b^All-polyethylene (n = 29) versus model-based metal-backed (n = 28).

## Discussion

We investigated whether model-based RSA, utilizing a different reference origin as compared with marker-based RSA, would affect migration outcomes. By doing so, we were also able to quantify movements of the polyethylene insert within the locking mechanism of the Triathlon metal tray and explore the disadvantages of each RSA method. If the results differed systematically, pooling and comparing RSA data from studies using different RSA techniques would be impaired unless adjusted for the methods being used. However, if the insert moves with respect to the metal tray in modular TKA, then marker-based migration values of the tibial component in the transverse plane are unreliable (Nilsson et al. 2003), and likely produce random error that cannot be corrected for when comparing with model-based RSA studies. Now that an increasing number of RSA studies are available with long-term follow-up, meta-analysis becomes possible—but one must ascertain pooling of data is justified when different RSA methods have been used.

Our study demonstrated systematic differences in translations but not rotations between model-based RSA and marker-based RSA. These differences are caused by the difference in reference origin that is used for migration calculation (Hurschler et al. 2009). As compared with the tibia 3D surface model, the origin in the center of the polyethylene markers overestimated the model-based transverse, longitudinal, and sagittal translations of the tibial component by 29%, 7%, and 26%, respectively. Correcting for this proportional bias, by using a factor or by using the same reference coordinate system in both analysis methods, resulted in nearly identical translations between model-based and marker-based analysis. For the rotations and MTPM values, the limits of agreement between marker-based and model-based RSA were larger because of the reduced precision of model-based rotations, particularly about the longitudinal axis. This is known and due to the relatively round, symmetrical shape of the tibial component in the transverse plane (Kaptein et al. 2007). Still, the limits of agreement between methods were within ± 0.5 mm and ± 0.8° and conclusions on the primary outcome of the RCT regarding group differences in MTPM remained unchanged. Furthermore, we found no evidence for the presence of insert micromotion and excluded this as a cause of unreliable marker-based migration measurements for the modular Triathlon TKA system. For the individual patient, however, use of a different method may result in substantial differences due to various types of measurement error (e.g., marker occlusion and model-fit inaccuracies). Therefore, one must not put too much weight on strict migration thresholds in individual patients (e.g., 0.2 mm of MTPM migration in the second postoperative year).

Our findings are in line with an earlier comparison between marker-based and model-based RSA (Hurschler et al. 2009). However, in that study, among other methodological differences, a uniplanar RSA setup was used resulting in marked differences in accuracy between “in-plane” and “out-of-plane” translations and rotations. In the present study we used a biplanar technique, and we did not find such a dichotomy in accuracy. Nevertheless, our findings further support their conclusion that model-based RSA can be used interchangeably with marker-based RSA, at least for the Triathlon TKA, provided that the same reference origin is used or corrected for using a factor when analyzing translations.

Previous studies evaluating insert micromotion relative to the metal tray in modular TKAs found small movements in Nuffield fixed-bearing TKAs (Corin Medical Ltd., UK) (Hansson et al. 2005) and NexGen fixed-bearing TKAs (Zimmer, USA) (Nilsson et al. 2003). In the latter study, these movements were closely examined and found to be greater in the transverse plane, which corresponds to the polyethylene–metal tray interface (Nilsson et al. 2003). This contrasts with our results and may be explained by the different designs of the locking mechanisms that were used. In a recent retrieval study of Łapaj et al. (2017), backside damage as a result of abrasion following micromotion of the polyethylene was found in designs with dovetail locking mechanisms, especially in the NexGen trays. Contrarily, they found no evidence for abrasion in the Triathlon knees owing to the full peripheral capture locking mechanism. Furthermore, the anti-rotational central island of the Triathlon design has been shown to effectively reduce micromotion to a minimum for a given reacted torque as compared with other TKA designs, including NexGen (Bhimji et al. 2010), although this mechanical study was performed by the research and development department of Stryker. It should be noted, however, that random error as a result of the reduced precision of model-based RSA limits firm conclusions on the presence of (longitudinal) rotations of the polyethylene within the locking mechanism. Nevertheless, the found translations were minimal and all outliers were found to be caused by polyethylene marker instability or occlusion, thus unlikely to be the result of micromotion in the polyethylene–metal tray interface.

A limitation of this study is that we compared the results of only one tibial component design. As the precision of model-based RSA depends on the shape and accuracy of the fitted model (Kaptein et al. 2003), differences between marker-based and model-based RSA results may be smaller or larger depending on the TKA design and also depending on the location of the prosthesis markers, either in the insert, or attached to the metal tibial component.

In summary, systematic differences in translations between marker-based and model-based RSA analysis disappeared when adjusted for the different reference origins being used for migration calculations. Micromotions of the polyethylene insert within the Triathlon metal tray were at most negligibly small. Mean migration results of model-based and marker-based measurements were comparable between groups when using the same reference origin, even though migration patterns of individual patients may differ between RSA methods due to various types of measurement error. 

### Supplementary data

Tables 3–4 and the Appendix are available as supplementary data in the online version of this article, http://dx.doi.org/10.1080/17453674.2019.1605692

The study was designed and coordinated by KH and STL. Data collection was performed by KH. Statistical analysis was done by KH, PM, and BK. KH, PM, LK, RN, STL, and BK interpreted the data and wrote the initial draft manuscript. All authors critically revised and approved the manuscript.

The authors are grateful to Håkan Leijon for providing the marker-based RSA measurements and for his valuable help in performing the additional model-based RSA measurements.  

*Acta* thanks Raed Itayem, Maiken Stilling and Lars Weidenhielm for help with peer review of this study.

## Supplementary Material

Supplemental Material
