# Supplementary material for: Marker-based versus model-based radiostereometric analysis of total knee arthroplasty migration: a reanalysis with comparable mean outcomes despite distinct types of measurement error
Source: Acta Orthop. 2019 Apr 24;90(4):366–72. doi: 10.1080/17453674.2019.1605692 (PMC7025693; doi:10.1080/17453674.2019.1605692)
Supplement: Supplemental Material [file IORT_A_1605692_SM0397.pdf]

Supplementary data

Table 3. RSA migration analysis of mean absolute translation and rotation along and about each orthogonal axis (log-values are back-transformed in the original scale)

	All-polyethylene mean (95% CI) (n = 29)	Metal-backed marker-based mean (95% CI) (n = 28)	Metal-backed model-based mean (95% CI) (n = 28)
Translation along transverse axis (mm)			
3 months	0.14 (0.09–0.20)	0.21 (0.15–0.27)	0.20 (0.15–0.27)
1 year	0.14 (0.09–0.20)	0.21 (0.16–0.27)	0.21 (0.16–0.27)
2 years	0.19 (0.14–0.25)	0.26 (0.20–0.32)	0.26 (0.20–0.32)
Translation along longitudinal axis (mm)			
3 months	0.12 (0.08–0.15)	0.11 (0.08–0.15)	0.12 (0.08–0.15)
1 year	0.13 (0.09–0.16)	0.13 (0.09–0.16)	0.15 (0.12–0.19)
2 years	0.10 (0.07–0.14)	0.15 (0.11–0.18)	0.17 (0.14–0.21)
Translation along sagittal axis (mm)			
3 months	0.19 (0.11–0.27)	0.22 (0.14–0.31)	0.24 (0.16–0.33)
1 year	0.24 (0.16–0.33)	0.38 (0.29–0.47)	0.38 (0.29–0.48)
2 years	0.25 (0.17–0.34)	0.44 (0.35–0.55)	0.43 (0.34–0.53)
Rotation about transverse axis (°)			
3 months	0.38 (0.27–0.49)	0.23 (0.14–0.34)	0.25 (0.15–0.35)
1 year	0.48 (0.37–0.61)	0.38 (0.27–0.49)	0.40 (0.30–0.52)
2 years	0.47 (0.36–0.59)	0.47 (0.35–0.59)	0.45 (0.33–0.57)
Rotation about longitudinal axis (degrees)			
3 months	0.18 (0.11–0.25)	0.19 (0.13–0.27)	0.29 (0.22–0.38)
1 year	0.20 (0.13–0.28)	0.24 (0.17–0.31)	0.38 (0.30–0.47)
2 years	0.20 (0.13–0.27)	0.28 (0.20–0.35)	0.41 (0.32–0.50)
Rotation about sagittal axis (°)			
3 months	0.26 (0.18–0.33)	0.24 (0.16–0.32)	0.21 (0.14–0.28)
1 year	0.32 (0.25–0.40)	0.28 (0.20–0.36)	0.24 (0.16–0.31)
2 years	0.34 (0.26–0.42)	0.33 (0.25–0.41)	0.25 (0.18–0.33)

Table 4. Adjusted RSA migration analysis of log-transformed maximum total point motion (logMTPM)

	Mean difference in logMTPM between groups (95% CI) Marker-based [a]	Model-based [b]
Treatment effect (reference: all-polyethylene)		
3 months	–0.007 (–0.049 to 0.036)	0.013 (–0.031 to 0.057)
1 year	0.014 (–0.029 to 0.057)	0.025 (–0.019 to 0.069)
2 years	0.030 (–0.013 to 0.074)	0.038 (–0.007 to 0.083)
Sex effect (reference: male)		
3 months	0.008 (–0.043 to 0.045)	0.002 (–0.044 to 0.047)
1 year	0.017 (–0.027 to 0.062)	0.011 (–0.034 to 0.057)
2 years	0.026 (–0.020 to 0.068)	0.031 (–0.015 to 0.077)
Surgeon effect (reference: surgeon 1)		
3 months	0.083 (0.040 to 0.126)	0.077 (0.033 to 0.121)
1 year	0.113 (0.071 to 0.156)	0.099 (0.055 to 0.143)
2 years	0.132 (0.089 to 0.174)	0.114 (0.070 to 0.158)

[a] All-polyethylene (n = 29) versus marker-based metal-backed (n = 28).
[b] All-polyethylene (n = 29) versus model-based metal-backed (n = 28).

Appendix

Prosthesis migration

RSA is generally used to calculate prosthesis migration, defined as the change in position and orientation of a prosthesis with respect to the bone (Valstar et al. 2005, ISO 16087:2013(E) 2013). Tantalum markers inserted into the bone and added to the prosthesis define landmarks that are used for accurate calculations. In Model-based RSA, the prosthesis itself is used as a marker, making prosthesis markers obsolete. By matching the virtual projections of a 3D surface model of the prosthesis with the detected roentgen projections of the prosthesis, the position and orientation of the prosthesis is calculated (Kaptein et al. 2003). First step in migration calculation is the landmark transform that aligns the bone markers in the follow-up moment (t1) with the bone markers in the reference moment (t0) (Valstar et al. 2005). This removes the "patient movement" between the different RSA acquisition moments (Figure A1).

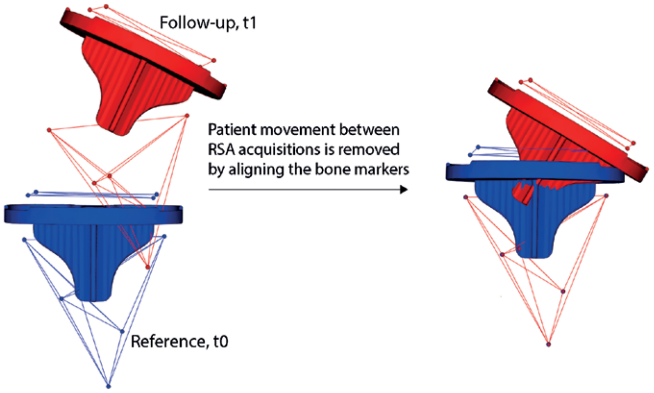

Figure A1. Transformation of the follow-up bone markers in the follow-up moment (t1) to the bone markers in the reference moment (t0) is performed (note that, in this example, the prosthesis migration is exaggerated).

The second step is the calculation of the change in position and orientation of the prosthesis between the reference moment and the follow-up moment. This change in position and orientation is thus relative to the bone markers.

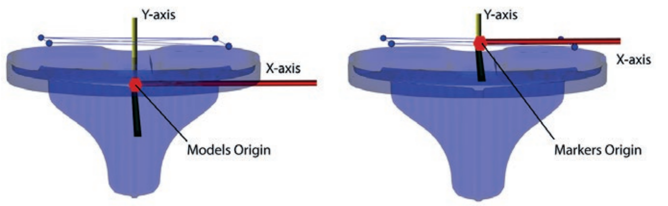

Figure A2. The left side of the figure shows the position of the reference origin of the 3D surface model (Model Origin) used for model-based RSA migration calculation and the right side of the figure shows the reference origin in the geometric center of the polyethylene markers (Markers Origin) used for marker-based RSA migration calculation. The X-axis is the transverse axis, the Y-axis is the longitudinal axis, and the Z-axis is the sagittal axis.

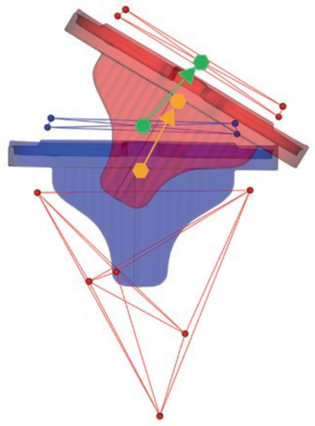

Figure A3. The prosthesis model migrated from t0 (blue) to t1 (red). The orange vector indicates the translation of the Model Origin in Model-based RSA. For the Markers Origin, translation is different (green vector).

In routine RSA calculations migration is expressed in a coordinate system that has its origin in the geometric center of either the prosthesis 3D surface model, or the prosthesis markers, in the reference follow-up moment, and is aligned with the global coordinate system as defined by the calibration cage of the reference RSA examination (Selvik 1989, Valstar et al. 2005, ISO 16087:2013(E) 2013). We call this coordinate system the reference coordinate system. In RSA calculations, the translation is calculated for the reference origin (Figure A2).

The calculated migration describes a transformation of the prosthesis from the reference moment to the follow-up moment and is expressed as a series of rotations about the 3 orthogonal axes and translations along these axes. The mathematics of RSA calculations are extensively described in Selvik (1989) and Söderkvist and Wedin (1993) and we will visually demonstrate the effect of changing the reference origin, without changing the orientation of the reference coordinate system, on the calculated migration (Figure 3).

Because the prosthesis in itself is a rigid structure (rigid body), the entire prosthesis rotates exactly the same from t0 to t1. Changing the reference origin position from "Model Origin" to "Markers Origin" and maintaining the orientation of the coordinate system does not affect the rotation of the prosthesis from t0 to t1. In Figure A3 the orange vector indicates the migration of the "Model Origin" in model-based RSA migration calculation and the green vector indicates the migration of the "Markers Origin" for marker-based RSA using polyethylene markers. The calculated translations along the orthogonal axes, for the Model and Markers reference origins, are different:

Calculated translations for the 2 reference origin positions (in simplified example):

Model Origin translation (x, y, z):	10.00	20.00	0.00
Markers Origin translation (x, y, z):	16.50	18.25	0.00

In Figure A3 these differences are reflected by different direction and length of the orange and green vectors.

In Figure A4 the effect of the position of the reference origin is shown in steps for the migration of the tibia prosthesis from t0 to t1. The position of the blue model after the Z-axis rotation differs slightly due to the difference of the reference origins: the upper row is for the Models Origin reference and the lower row is for the Markers Origin reference.

Point motion, maximum total point motion

For individual points on the prosthesis (e.g., markers attached to the prosthesis, virtual markers or 3D surface model points) the translation along each axis can be calculated from the x-, y-, and z-coordinates of these points at t1 and t0. The point motion can be calculated based on Pythagoras' theorem:

$$\text{point motion} = \sqrt{(Tx^2 + Ty^2 + Tz^2)}$$

In Figure A5 the point motion of 4 virtual markers on the tibia prosthesis is shown.

The point motion of the virtual markers from Figure A5 is:

	x	y	z	Point motion (mm)
Front	10.41	19.89	0	22.45
Lateral	15.56	39.12	0	42.10
Medial	5.26	0.67	0	5.30
Tip	–6.15	24.33	0	25.09

The virtual marker with the largest point motion is the "Lateral" marker. The virtual marker with the smallest point motion is the "Medial" marker. In the example migration shown in this Appendix, the tibia model rotates approximately around the medial edge of the prosthesis. Virtual markers close to this "true" rotation point have small point motions, and virtual markers at larger distances from this true rotation point have larger point motions. Maximum total point motion (MTPM), which is frequently used to summarize the migration of a prosthesis, is the length of the translation vector of the marker or virtual marker in a rigid body that has the greatest migration. For model-based RSA, MTPM is the length of the translation vector of the point on the model that moved the most.

The difference between the x-, y-, and z-coordinates at t0 and t1 used to calculate point motion is independent of the selected reference origin. As a consequence, point motion, including MTPM, will not differ between migration calculations with different reference origins.

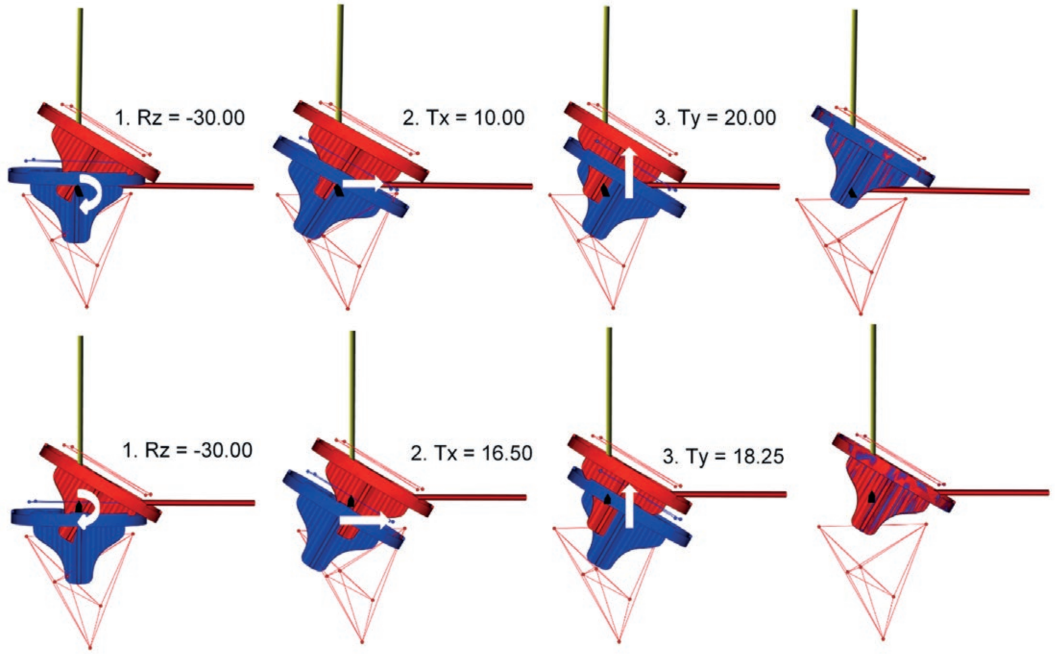

Figure A4. The upper row illustrates RSA migration of the tibia prosthesis from t0 (blue) to t1 (red) using the Model Origin: the model is rotated about the Z-axis (–30°), and translated along the X-axis (10 mm) and Y-axis (20 mm). The lower row illustrates RSA migration of the tibia prosthesis from t0 (blue) to t1 (red) using the Markers Origin: the model is rotated about the Z-axis (–30°), and translated along the X-axis (16.5 mm) and Y-axis (18.25 mm).

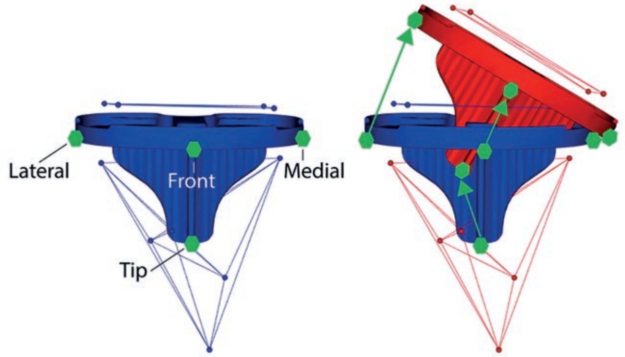

Figure A5. The change in position of 4 virtual markers on the tibia prosthesis model from t0 (blue) to t1 (red)

In summary

- The position of the reference coordinate system, used to describe prosthesis migration, has an effect on the calculated prosthesis translations but not on the prosthesis rotations.
- The translation of individual markers, virtual markers, or points on the 3D surface model are not affected by the position of the reference origin. Hence, MTPM is not affected by changing the reference origin.
- In general it can be stated that the further away from the true point of rotation a (virtual) marker lies, the larger the calculated translations are. This also applies to the reference origin, as this is also a "point."
- Changing the orientation of the reference coordinate system (not demonstrated in this Appendix) does have an effect on the calculated translations and rotations of the prosthesis.
- Changing the orientation of the reference coordinate system does not affect the magnitude of individual point motion, but it does affect the direction of the point motion.

Appendix references

ISO 16087:2013(E). Implants for surgery: Roentgen stereophotogrammetric analysis for the assessment of migration of orthopaedic implants. Geneva: International Organization for Standardization; 2013.

Kaptein B L, Valstar E R, Stoel B C, Rozing P M, Reiber J H. A new model-based RSA method validated using CAD models and models from reversed engineering. J Biomech 2003; 36(6): 873-82.

Selvik G. Roentgen stereophotogrammetry: a method for the study of the kinematics of the skeletal system. Acta Orthop Scand Suppl 1989; 232: 1-51.

Söderkvist I, Wedin P A. Determining the movements of the skeleton using well-configured markers. J Biomech 1993; 26(12): 1473-7.

Valstar E R, Gill R, Ryd L, Flivik G, Borlin N, Karrholm J. Guidelines for standardization of radiostereometry (RSA) of implants. Acta Orthop 2005; 76(4): 563-72.
